# Cell Type-Specific Membrane Potential Changes in Dorsolateral Striatum Accompanying Reward-Based Sensorimotor Learning

**DOI:** 10.1093/function/zqab049

**Published:** 2021-09-21

**Authors:** Tanya Sippy, Corryn Chaimowitz, Sylvain Crochet, Carl C H Petersen

**Affiliations:** Laboratory of Sensory Processing, Brain Mind Institute, Faculty of Life Sciences, Ecole Polytechnique Fédérale de Lausanne (EPFL), Lausanne, CH-1015, Switzerland; Department of Psychiatry and Physiology and Neuroscience, New York University Langone Medical Center, New York, NY 10016, USA; Department of Psychiatry and Physiology and Neuroscience, New York University Langone Medical Center, New York, NY 10016, USA; Laboratory of Sensory Processing, Brain Mind Institute, Faculty of Life Sciences, Ecole Polytechnique Fédérale de Lausanne (EPFL), Lausanne, CH-1015, Switzerland; Laboratory of Sensory Processing, Brain Mind Institute, Faculty of Life Sciences, Ecole Polytechnique Fédérale de Lausanne (EPFL), Lausanne, CH-1015, Switzerland

**Keywords:** striatum, goal-directed sensorimotor transformation, reward-based learning, whole-cell membrane potential

## Abstract

The striatum integrates sensorimotor and motivational signals, likely playing a key role in reward-based learning of goal-directed behavior. However, cell type-specific mechanisms underlying reinforcement learning remain to be precisely determined. Here, we investigated changes in membrane potential dynamics of dorsolateral striatal neurons comparing naïve mice and expert mice trained to lick a reward spout in response to whisker deflection. We recorded from three distinct cell types: (i) direct pathway striatonigral neurons, which express type 1 dopamine receptors; (ii) indirect pathway striatopallidal neurons, which express type 2 dopamine receptors; and (iii) tonically active, putative cholinergic, striatal neurons. Task learning was accompanied by cell type-specific changes in the membrane potential dynamics evoked by the whisker deflection and licking in successfully-performed trials. Both striatonigral and striatopallidal types of striatal projection neurons showed enhanced task-related depolarization across learning. Striatonigral neurons showed a prominent increase in a short latency sensory-evoked depolarization in expert compared to naïve mice. In contrast, the putative cholinergic striatal neurons developed a hyperpolarizing response across learning, driving a pause in their firing. Our results reveal cell type-specific changes in striatal membrane potential dynamics across the learning of a simple goal-directed sensorimotor transformation, helpful for furthering the understanding of the various potential roles of different basal ganglia circuits.

## Graphical Abstract

**Graphical Abstract:**
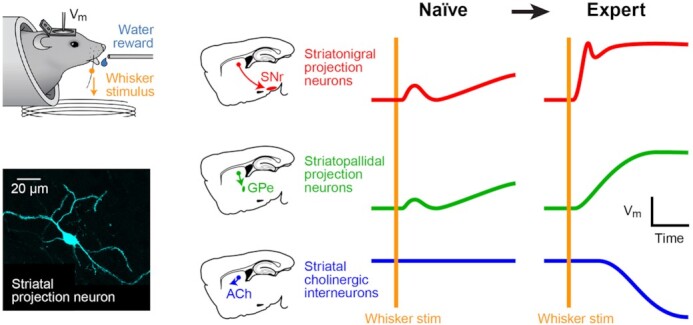

## Introduction

The changes in neural circuits underlying reward-based sensorimotor learning remain incompletely understood. The dorsolateral striatum (DLS) is thought to be critically involved, as it receives sensorimotor inputs from thalamus and cortex, and sends its outputs to downstream basal ganglia nuclei important for motor control.^1–5^ The DLS is also a major target of dopaminergic innervation, which might serve important functions for reward-based learning,^6–9^ including through the differential regulation of synaptic plasticity in specific types of striatal neurons.^2,3,10–15^ Although striatal synaptic plasticity could therefore underlie important aspects of sensorimotor learning,^12,16–18^ this hypothesis has not yet been tested by measuring cell type-specific changes in membrane potential (V_m_) dynamics across reward-based learning.

In a previous study, we found that V_m_ dynamics of striatal projections neurons (SPNs) in whisker-related DLS were strongly modulated during performance of a task in which mice were trained to lick a water-reward spout in response to a whisker deflection.^19^ Here, in a new set of recordings, we compare V_m_ responses from naïve and expert mice, before and after task learning. We find increased task-related depolarizing responses in anatomically and genetically-identified types of SPNs across learning. We also recorded tonically active, putative cholinergic, interneurons (TANs), which form a small distinct population of cells with large somata in the striatal microcircuitry.^20–23^ TANs developed a hyperpolarizing response and a pause in action potential firing with task learning, in agreement with previous studies.^24–26^ Our data are consistent with the hypothesis that prominent cell-type-specific changes in striatal activity might accompany reward-based sensorimotor learning.

## Materials and Methods

### Mice

All experiments were carried out with male and female mice in accordance with the Swiss Federal Veterinary Office (authorization VD1628.6). Mice were 4–7 weeks old at the time of implantation and 6–12 weeks old at the time of recordings. Adora2a-Cre mice (MMRRC: 036158-UCD) and Drd1a-Cre mice (MMRRC: 030778-UCD) were crossed with Lox-Stop-Lox-tdTomato mice (JAX: 007909). Drd1a-tdTomato (JAX: 016204) were crossed with Drd2-Green Fluorescent Protein (GFP) (MGI: 3843608). The mice were implanted with a light-weight metal head-post and a recording chamber under ketamine/xylazine anesthesia. The position of the DLS was stereotaxically marked at the surface of the skull (0 mm anterior and 2.8–3.0 mm lateral of bregma). After the surgery, the animals were returned to their home cage for 5–7 days of recovery. The mice were housed in groups of 2–4 mice with a reverse light/dark cycle (light 7 pm to 7 am), at a temperature of 22 ± 2°C with food available ad libitum.

### Behavior

**Figure 1.**
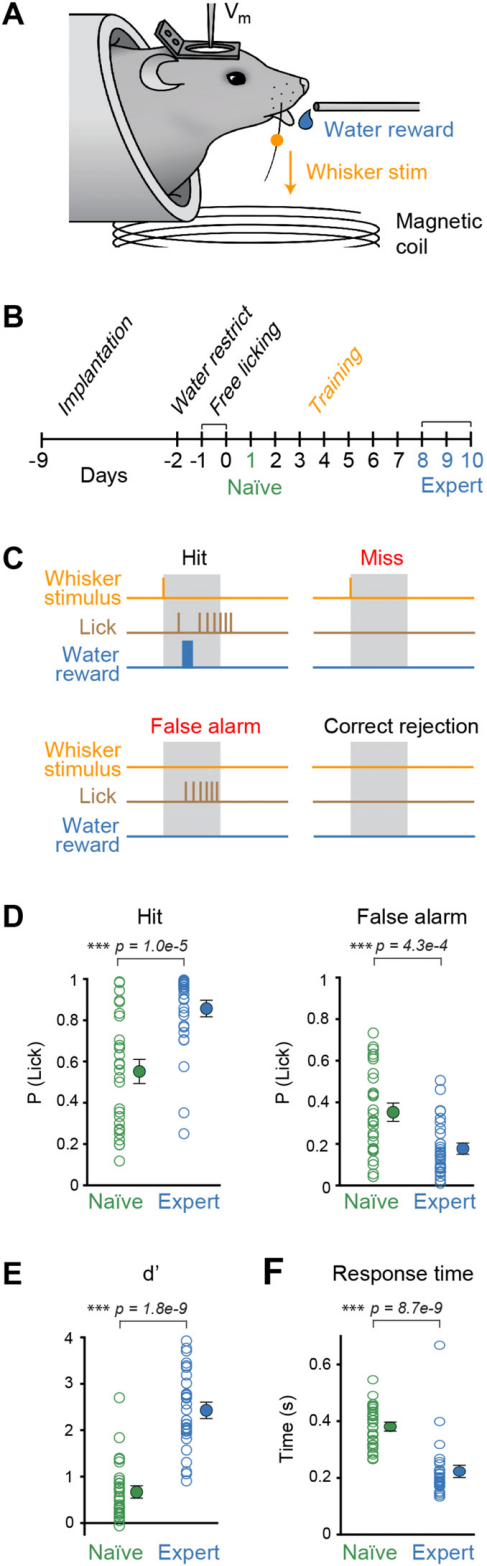
Whisker detection task. (A) Depiction of the whisker-based sensory detection task: mice learned to associate a brief (1 ms) downward deflection of their right C2 whisker with the availability of a water reward. (B) Whole-cell (V_m_) recordings were performed in the DLS of head-restrained mice during the first training session (day 1) of this task (naïve, green) or in mice that had been trained for 7 or more days (expert, blue). (C) Trials were classified as hit if the mouse licked within the 1 s response window that followed whisker stimulus (grey area), as miss if the mouse did not lick, as false alarm if it licked when no whisker stimulus was presented (catch trials) and as correct rejection if it did not lick on catch trials. Stimulus and catch trials were randomly interleaved and separated by a randomized 4–12 s inter-trial interval. In addition, the mouse was required to not lick in the 4 s before a trial was initiated to prevent compulsive licking. (D) The probability of licking in the response window of naïve (n = 26 mice, green) and expert (n = 20 mice, blue) mice during the V_m_ recordings in trials with a whisker stimulus (hit rate, left) or catch trials without a whisker stimulus (false alarm rate, right) (Wilcoxon–Mann–Whitney test). Open circles indicate individual cells, closed circles with error bars indicate mean ± standard error of mean (SEM). (E) The discriminability (d') of trials with and without whisker stimuli compared between naïve and expert mice (Wilcoxon–Mann–Whitney test). Open circles indicate individual cells, closed circles with error bars indicate mean ± SEM. (F) Response time of naïve and expert mice (Wilcoxon-Mann-Whitney test). Open circles indicate individual cells, closed circles with error bars indicate mean ± SEM.

One week after implantation, all whiskers were trimmed except the C2 whisker on either side. After 1–2 days of adaption to head-restraint, mice were water restricted to 1 ml of water/day and the mice were trained in a whisker detection task, as previously described.^19^ Training started with 2 days of free-licking during which mice were habituated to trigger the reward delivery by licking the water spout, but no whisker stimulus was delivered. Following the two free-licking sessions, mice were trained in the sensory detection task. At the beginning of each training session, a small (2 mg) metal particle was attached to the right C2 whisker allowing the whisker to be vertically deflected by a 1 ms current pulse passed through an electromagnetic coil placed immediately beneath the head of the mouse (Figure 1A and B). Ambient white noise (80 dB) was played at all times to mask any potential auditory cues arising from whisker stimulation. Mice were trained to associate the C2 whisker deflection with the availability of water at the reward spout. If the mouse licked the spout within the reward window (1 s), it was considered a hit trial, and the mouse received a drop of water. If not, it was considered a miss trial and no reward was delivered. Whisker stimuli were delivered without any preceding cues at random time intervals, with intertrial interval ranging between 4 and 12 seconds. To discourage spontaneous licking, a 4-s no-lick period was imposed during which no lick should occur in order to start a trial. Trials with whisker stimuli were randomly interleaved with catch trials in which no stimulus was given. If licks occurred during the response window of a catch trial, it was considered a false alarm, if not it was considered a correct rejection. After a few training days (8–10 days), mice were able to achieve a stable level of performance, with a high hit rate and a low false alarm rate, and were then considered as expert. Electrophysiological recordings were performed either during the first training session of the sensory detection task (naïve—i.e., the first time the mice were exposed to the whisker stimulus) or once the mice had reached good performance (expert). Naïve and expert mice were non-overlapping in order to facilitate the anatomical identification of recorded neurons.

### Electrophysiology

Whole-cell patch-clamp recording electrodes (6–8 MΩ) were filled with an intracellular solution containing (in mM) 135 potassium gluconate, 4 Potassium chloride, 10 HEPES, 10 sodium phosphocreatine, 4 MgATP, and 0.3 Na_3_GTP (adjusted to pH 7.3 with KOH), to which 3–5mg/ml biocytin was added. V_m_ was recorded using a Multiclamp 700B amplifier without injection of holding current and was not corrected for liquid junction potentials. On the day of recording, a small (less than 0.5 mm diameter) craniotomy was made under isoflurane anesthesia over the DLS. Mice were allowed to recover from anesthesia for 2–4 hours. Then, whole-cell patch-clamp recordings were obtained as previously described.^19,27,28^ At the start of each recording, a series of increasing current steps was injected into each neuron. We proceeded with the recording if the neuron displayed both a stable resting V_m_ and overshooting action potentials. At the end of the recording session, mice were transcardially perfused with Phosphate buffered saline and paraformaldehyde (4%) solutions and the brain was removed for anatomy. Using a vibratome, 100 μm-thick coronal brain sections were cut, and stained with streptavidin coupled to Alexa 647 (1:2000, Invitrogen) to reveal biocytin filling of postsynaptic neurons. Confocal imaging was used to evaluate co-localization of the biocytin-labelled soma of the recorded neuron with the fluorescent protein indicating the genetically-defined cell class. Low magnification fluorescence imaging was used to image the neuron in the context of the entire brain slice. These images were then loaded into Fiji software and the coordinates of labelled cells calculated using built-in software tools. The anterior posterior coordinate was estimated by matching the anatomical markers in the bright field image of the slice with a mouse brain atlas.^29^

### Quantification and Statistical Analysis

All data analysis was performed in MATLAB using custom written algorithms. To assess the whisker stimulus-triggered response, V_m_ changes were evaluated relative to a baseline V_m_ averaged over the 100ms before the whisker stimulus. To obtain the lick-triggered average, V_m_ traces were aligned to the time of the first tongue-spout contact in a bout of licking with a baseline period of 500–200 ms before the first lick time. All values are presented as mean ± SEM. Non-parametric statistical tests were used to assess significant differences. The Wilcoxon–Mann–Whitney 2-sample rank test was used for unpaired samples (naïve vs expert). The Wilcoxon signed rank test was used for paired samples (hit vs miss trials, with a minimum number of 2 trials of each type for inclusion of neurons in this analysis). Bonferroni correction was applied for comparison between the 3 cell types.

## Results

### Reward-Based Learning of A Whisker Detection Task

We recorded from neurons located in the left DLS before and after mice learned a whisker-based sensory detection task (Figure 1A and B). Mice received a water reward if they licked a spout within 1 second after a brief (1 ms) single deflection of the right C2 whisker. Catch trials were randomly interleaved, in which no stimulus was applied, in order to assess false alarm rate (Figure 1C). Trials occurred at randomized 4–12 s intervals. Performance in expert mice (n = 20 mice) was significantly better than in naïve mice (n = 26 mice) with an increased hit rate (expert hit rate 86 ± 3%, n = 30 recordings; naïve hit rate 55 ± 5%, n = 29 recordings; Wilcoxon–Mann–Whitney *P* = 1.0 × 10^–5^),  and a decreased false alarm rate (expert false alarm rate 18 ± 2%, n = 30 recordings; naïve false alarm rate 35 ± 4%, n = 29 recordings; Wilcoxon–Mann–Whitney *P* = 4.3 × 10^–4^) (Figure 1D). Discriminability (d’) of trials with and without whisker stimulus therefore increased (expert d’ 2.4 ± 0.1, n = 30 recordings; naïve d’ 0.7 ± 0.1, n = 29 recordings; Wilcoxon–Mann–Whitney *P* = 1.8 × 10^–9^) (Figure 1E). Response times were shorter in expert compared to naïve mice (expert 223 ± 18 ms, n = 30 recordings; naïve 381 ± 13 ms, n = 29 recordings; Wilcoxon–Mann–Whitney *P* = 8.7 × 10^–9^) (Figure 1F).

### Intrinsic Properties of Striatonigral, Striatopallidal, and Putative Cholinergic Striatal Neurons

**Figure 2.**
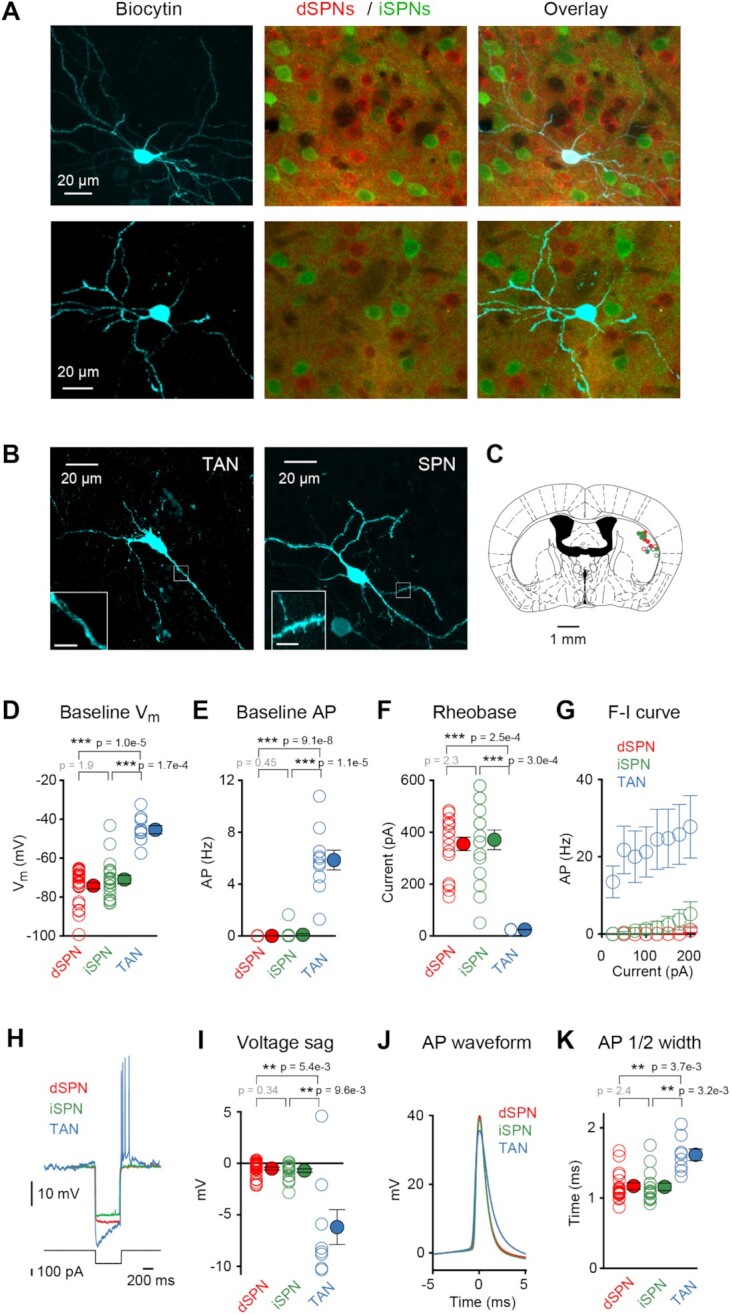
Cell-type identification. (A) Fluorescent biocytin staining (cyan) of 2 example neurons recorded in the DLS of two different genetically-engineered mice expressing tdTomato in dSPNs (red) and Green Fluorescent Protein (GFP) in iSPNs (green). In the top example cell, the biocytin signal colocalizes with tdTomato revealing this neuron to be a dSPN. In the bottom example cell, biocytin signal colocalizes with GFP revealing this neuron to be an iSPN. (B) Left: example of a biocytin-labelled aspiny TAN. Right: example of a biocytin filled spiny SPN. Inset scale bar 5 µm. (C) Example coronal drawing (AP −0.22 mm)^29^ showing cell locations of dSPNs (red), iSPNs (green), and TANs (blue) recorded in naïve (open circles) or expert (filled circles) mice. (D–F) The baseline V_m_ (D), baseline action potential (AP) firing rate (E) and rheobase (F) of dSPNs, iSPNs, and TANs combining both naïve and expert mice (comparison between dSPN vs iSPN, dSPN vs TAN, and iSPN vs TAN, Wilcoxon–Mann–Whitney test with Bonferroni correction). (G) Grand average action potential firing rates versus the amount of current injected (F–I curves) for dSPNs, iSPNs, and TANs combining both naïve and expert mice. (H) Example V_m_ traces from a dSPN, an iSPN, and a TAN during a hyperpolarizing current step, demonstrating a voltage sag in the TAN. (I) Quantification of the voltage sag of dSPNs, iSPNs, and TANs combining both naïve and expert mice (comparison between dSPN vs iSPN, dSPN vs TAN, and iSPN vs TAN, Wilcoxon–Mann–Whitney test with Bonferroni correction). (J) Action potential waveforms for dSPNs, iSPNs, and TANs. (K) Action potential half-width for dSPNs, iSPNs, and TANs combining both naïve and expert mice (comparison between dSPN vs iSPN, dSPN vs TAN, and iSPN vs TAN, Wilcoxon–Mann–Whitney test with Bonferroni correction). See also Supplementary Figures 1 and 2.

We targeted whole-cell recordings^30^ to regions of the DLS known to receive input from primary whisker-related somatosensory cortex,^19,27,31–34^ and compared V_m_ across naïve and expert mice during task performance. The DLS is composed of different types of neurons, and, in this study, we differentiated between dopamine receptor type 1-expressing direct pathway striatonigral neurons (dSPNs), dopamine receptor type 2-expressing indirect pathway striatopallidal neurons (iSPNs) and TANs (putative cholinergic interneurons). The patch recording pipette contained biocytin, allowing for fluorescent post hoc staining and co-localization with tdTomato or GFP in mice engineered to specifically express these proteins in dSPNs and iSPNs^35,36^ (Figure 2A). For SPNs, we only included anatomically identified neurons which were co-labelled with fluorescent proteins to positively characterize striatonigral and striatopallidal projection neurons (Supplementary Table 1). TANs had aspiny dendrites compared with SPNs (Figure 2B), and were readily identified during recording because of their distinct electrophysiological properties.^23,27,37^ The somata of the recorded neurons across naïve and expert mice were located in a similar region of the DLS (Figure 2C and Supplementary Figure 1), previously revealed to be innervated by whisker-sensory cortex .^19^

Relative to SPNs, TANs had a more depolarized baseline V_m_ (baseline V_m_ dSPN −74.2 ± 1.5 mV, n = 29 cells; baseline V_m_ iSPN −71.0 ± 2.2 mV, n = 20 cells; baseline V_m_ TAN −45.4 ± 2.1 mV, n = 10 cells; Wilcoxon–Mann–Whitney test with Bonferroni correction dSPN vs iSPN *P* = 1.9, dSPN vs TAN *P* = 1.0 × 10^–5^, and iSPN vs TAN *P* = 1.7 × 10^–4^) (Figure 2D). Baseline V_m_ did not differ comparing naïve and expert mice for dSPNs, iSPNs, or TANs (Supplementary Figure 2).

TANs had higher baseline action potential firing rates (baseline firing rate dSPN 0.002 ± 0.001 Hz, n = 29 cells; baseline firing rate iSPN 0.09 ± 0.08 Hz, n = 20 cells; baseline firing rate TAN 5.8 ± 0.8 Hz, n = 10 cells; Wilcoxon–Mann–Whitney with Bonferroni correction dSPN vs iSPN *P* = 0.45, dSPN vs TAN *P* = 9.1 × 10^-8^,  and iSPN vs TAN *P* = 1.1 × 10^–5^) (Figure 2E). Within the limited dataset, baseline firing rates were overall relatively similar comparing across naïve and expert mice (Supplementary Figure 2).

TANs were more easily excited by injection of depolarizing current compared to SPNs (Figure 2F and G), as reported previously.^38^ Rheobase (minimal depolarizing current needed to evoke action potential firing) did not differ across learning for SPNs (Supplementary Figure 2). We did not compare rheobase across learning for TANs, since these neurons were spontaneously active in both naïve and expert mice.

TANs had a characteristic voltage sag (voltage sag dSPN −0.5 ± 0.1 mV, n = 28 cells; voltage sag iSPN −0.7 ± 0.2 mV, n = 19 cells; voltage sag TAN −6.2 ± 1.7 mV, n = 8 cells; Wilcoxon–Mann–Whitney with Bonferroni correction dSPN vs iSPN p = 0.34, dSPN vs TAN p = 0.0054, and iSPN vs TAN p = 0.0096) (Figure 2H and I), and broader action potentials (half-width dSPN 1.2 ± 0.1 ms, n = 17 cells; half-width iSPN 1.2 ± 0.1 ms, n = 16 cells; half-width TAN 1.6 ± 0.1 ms, n = 8 cells; Wilcoxon–Mann–Whitney with Bonferroni correction dSPN vs iSPN *P* = 2.4, dSPN vs TAN *P* = 0.0037, and iSPN vs TAN *P* = 0.0032) (Figure 2J and K).

### Cell Type-Specific V_m_ Dynamics Across Task Learning

**Figure 3.**
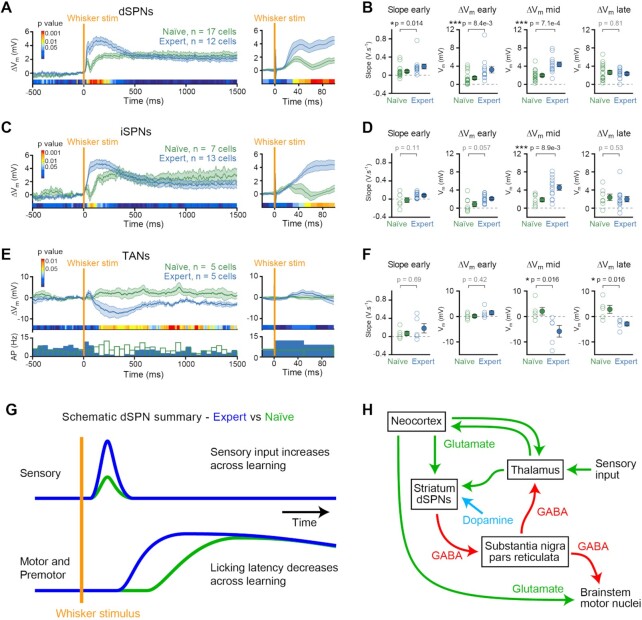
Learning is accompanied by cell-type specific changes in membrane potential dynamics aligned to the time of whisker stimulation. (A) Left: Whisker-stimulus triggered V_m_ average for hit trials in dSPNs recorded from naïve mice (green, n = 17 cells) and expert mice (blue, n = 12 cells). Right: higher temporal resolution enabling visualization of the early response. The color-coded bar indicates *P*-values for the difference between expert and naïve mice in 10 ms time windows (Wilcoxon–Mann–Whitney test). (B) The early slope (20–30 ms after stimulus), early ΔV_m_ (20–50 ms after stimulus), mid ΔV_m_ (50–250 ms after stimulus), and late ΔV_m_ (0.5–1 s after stimulus) in dSPN naïve vs expert mice (Wilcoxon–Mann–Whitney test). (C) Left: whisker stimulus triggered V_m_ average for hit trials in iSPNs recorded from naïve mice (n = 7 cells) and expert mice (n = 13 cells). Right: higher temporal resolution. The color-coded bar indicates *P*-values for the difference between expert and naïve mice in 10 ms time windows (Wilcoxon–Mann–Whitney test). (D) The early slope (20–30 ms after stimulus), early ΔV_m_ (20–50 ms after stimulus), mid ΔV_m_ (50–250 ms after stimulus), and late ΔV_m_ (0.5–1 s after stimulus) in iSPNs from naïve vs expert mice (Wilcoxon–Mann–Whitney test). (E) Left: whisker stimulus triggered V_m_ average for hit trials in TANs recorded from naïve mice (n = 5 cells) and expert mice (n = 5 cells). Right: higher temporal resolution. The color-coded bar indicates *P*-values for the difference between expert and naïve mice in 10 ms time windows (Wilcoxon–Mann–Whitney test). (F) The early slope (20–30 ms after stimulus), early ΔV_m_ (20–50 ms after stimulus), mid ΔV_m_ (100–400 ms after stimulus), and late ΔV_m_ (0.5–1 s after stimulus) in TANs from naïve vs expert mice (Wilcoxon–Mann–Whitney test). (G) Schematic summary of the learning-related changes in dSPNs. The early sensory response evoked by whisker stimulation increases in dSPNs across learning. The later component of the response likely relates to motor and premotor inputs to dSPNs, which occur earlier in expert mice, since they lick with shorter latency. These two changes could account for the overall change in V_m_ dynamics of dSPNs across learning shown in panels A and B. (H) Whisker deflection will drive neurons in cortex and thalamus to release glutamate onto neurons in the DLS. During task learning and execution, the mouse receives a reward in hit trials upon licking after the whisker stimulus, which likely causes a transient increase in dopamine concentration. Increased dopamine could contribute to promoting long-term potentiation of synaptic input onto the D1R-expressing dSPNs. Enhanced sensory-evoked glutamatergic responses in dSPNs from presynaptic thalamic or cortical neurons could increase the probability of licking through inhibition of neurons in substantia nigra pars reticulata, which contains tonically active inhibitory neurons. This might result in disinhibition of motor thalamus and brainstem motor nuclei, thus contributing to driving licking as a motor response to whisker deflection after reward-based learning. See also Supplementary Figures 3 and 4.

Analysing hit trials of the whisker detection task, we found that both dSPNs and iSPNs had an enhanced whisker stimulus-evoked depolarization in expert mice compared to naïve mice (Figure 3A–D). The slope of the early sensory-evoked depolarization was significantly larger in expert mice compared to naïve mice for dSPNs (expert dSPNs slope 0.19 ± 0.05 V.s^–1^, n = 12 cells; naïve dSPNs slope 0.08 ± 0.02 V.s^–1^, n = 17 cells; Wilcoxon–Mann–Whitney naïve vs expert, *P* = 0.014) (Figure 3B, far left). The amplitude of the early depolarisation quantified 20–50 ms after the whisker stimulus was also significantly larger in expert mice compared to naïve mice for dSPNs (ΔV_m_ early, expert dSPNs ΔV_m_ 3.2 ± 0.7 mV, n = 12 cells; naïve dSPNs ΔV_m_ 1.4 ± 0.3 mV, n = 17 cells; Wilcoxon–Mann–Whitney naïve vs expert, *P* = 0.0084) (Figure 3B, center left). The negative response slopes and amplitudes in some recordings might result from inhibitory synaptic input or spontaneous noisy V_m_ fluctuations. Consistent with a previous study (Sippy et al., 2015), the early response in dSPNs of expert mice was significantly enhanced in hit trials compared to miss trials (Supplementary Figure 3) and the slope of the early response of expert mice was significantly faster in dSPNs than iSPNs (Wilcoxon–Mann–Whitney dSPN vs iSPN, *P* = 0.018).

After this initial sensory response there was a significantly enhanced depolarization in hit trials for expert mice compared to naïve mice in both dSPNs and iSPNs (ΔV_m_ mid, quantified 50–250 ms after the whisker stimulus: expert dSPNs ΔV_m_ 4.4 ± 0.5 mV, n = 12 cells; naïve dSPNs ΔV_m_ 2.0 ± 0.3 mV, n = 17 cells; Wilcoxon–Mann–Whitney naïve vs expert, *P* = 7.1 × 10^–4^;  expert iSPNs ΔV_m_ 4.5 ± 0.6 mV, n = 13 cells; naïve iSPNs ΔV_m_ 1.8 ± 0.4 mV, n = 7 cells; Wilcoxon–Mann–Whitney naïve vs expert, *P* = 0.0089) (Figure 3B and D, center right). The shorter reaction time after learning likely contributes to this enhanced secondary excitation of SPNs in expert mice, since the first lick in a bout of spontaneous licking is accompanied by a similar depolarization of dSPNs and iSPNs in both naïve and expert mice (Supplementary Figure 4). At longer post-stimulus times, there were no significant differences in the depolarization of dSPNs or iSPNs comparing naïve and expert mice (Figure 3B and D, far right). During this time period in hit trials, both groups of mice were licking to receive water, and the sustained depolarization might at least in part reflect this motor activity (Supplementary Figure 4).

The V_m_ dynamics of TANs were very different from the SPNs (Figure 3E–F). The grand average of the hit trial V_m_ responses in TANs revealed a pronounced, significantly larger hyperpolarization in expert mice compared to naïve mice (quantified across 100–400 ms after the whisker stimulation: expert TANs ΔV_m_ −5.8 ± 2.3 mV, n = 5 cells; naïve TANs ΔV_m_ 2.1 ± 1.5 mV, n = 5 cells; Wilcoxon–Mann–Whitney naïve versus expert, *P* = 0.016). The hyperpolarization of the V_m_ was accompanied by a significant decrease in firing rate following whisker stimulation for expert mice compared to naïve mice (quantified across 100–400 ms after the whisker stimulation: expert change in firing rate −5.8 ± 1.8 Hz, n = 5 cells; naïve change in firing rate 4.4 ± 1.8 Hz, n = 5 cells; Wilcoxon–Mann–Whitney naïve versus expert *P* = 0.03). The hyperpolarization observed in TANs in expert mice appeared to occur after a delay, and the early response amplitude (quantified 20–50 ms after the whisker stimulus) did not differ significantly comparing naïve and expert mice (Figure 3F).

## Discussion

Here, using the whole-cell recording technique, we examined V_m_ dynamics of 3 cell types in the striatum before and after learning a simple goal-directed sensorimotor transformation, finding 2 important changes: (i) SPNs in expert mice showed an enhanced depolarization compared to naïve mice; and (ii) TANs developed a hyperpolarizing response in expert mice.

### Enhanced Depolarization of SPNs in Expert Mice

We found that whisker deflection evoked depolarizing responses which were transiently larger in expert compared to naïve mice for both dSPNs and iSPNs in hit trials (Figure 3).^39^ Increased synchronous excitatory synaptic input across sensorimotor learning could drive the increased depolarization in expert mice. The reduced reaction time for licking in expert mice is likely to contribute (Figure 3G), since licking was associated with depolarization of SPNs (Supplementary Figure 4). Licking, planning to lick, and other movements, typically correlate with increased action potential firing of some cortical and thalamic neurons,^40–44^ part of which are likely corticostriatal and thalamostriatal neurons thus potentially directly driving depolarization of SPNs through increased release of glutamate (quantified in mid and late periods 50–250 ms and 500–1000 ms after whisker stimulation, respectively).

The very earliest depolarization (quantified 20–50 ms after whicker stimulation) occurs before movement initiation, and therefore represents the processing of sensory input and the decision to initiate licking. In this early time window after whisker deflection, we found a significant enhancement of the fast sensory-evoked depolarization of dSPNs across learning (Figure 3A, B, and G). Mechanistically, an increased excitation of dSPNs across learning could contribute to the previously-reported larger early depolarization in dSPNs compared to iSPNs in expert mice carrying out a similar whisker detection task.^19^ The baseline V_m_ and excitability did not change across learning (Supplementary Figure 2), suggesting that this change could result from increased glutamatergic synaptic input onto dSPNs. Dopamine activation of D1Rs has been shown to enhance long-term synaptic potentiation of glutamatergic synapses.^13,15^ Reward-related increases in DLS dopamine levels during hit trials^6–8,45^ might thus contribute to enhancing fast whisker deflection-evoked glutamatergic input on D1R-expressing dSPNs during learning (Figure 3G). Increased responses in dSPNs across learning could contribute to task execution by enhancing the inhibition of postsynaptic neurons in substantia nigra pars compacta (Figure 3H), consistent with the result that brief optogenetic excitation of dSPNs is sufficient to evoke licking in trained mice^19^. Our data therefore support a potential role for dopamine acting on D1R-expressing dSPNs contributing to reward-based learning, but further measurements and manipulations considering the many neuronal circuits and neuromodulatory systems of the basal ganglia are necessary to obtain a more complete understanding.

### Enhanced Hyperpolarization of TANs Across Learning

Cholinergic interneurons (TANs), despite being a small minority of local neurons in the DLS, are thought to play important roles in controlling the activity of SPNs^46^ and behavior.^47,48^ TANs have been shown to exhibit a pause in their firing in response to stimuli that trigger a learned and rewarded motor output.^24–26^ Here, we find a similar activity pattern following the learning of a whisker-dependent goal-directed sensorimotor transformation. Underlying this pause in firing is a hyperpolarization of the V_m_ of TANs (Figure 3). Synaptically, the hyperpolarization might result from local inhibitory circuits within the striatum.^49–54^ Long-range inhibitory input to TANs might also contribute, for example, midbrain dopaminergic and GABAergic neurons in substantia nigra and ventral tegmental area have been reported to innervate striatal TANs causing hyperpolarisation.^50,55–58^ Future experiments should therefore investigate the possible contributions of diverse synaptic circuits comprising different presynaptic neurons innervating TANs.

The pause in TAN firing presumably leads to a transient reduction in acetylcholine, which might have many diverse effects upon striatal neurons and synaptic transmission in the striatum,^59,60^ including through nicotinic receptors,^61,62^ presynaptic muscarinic receptors affecting neurotransmitter release,^63–67^ and postsynaptic muscarinic receptors affecting various ionic conductances.^68–70^ Future pharmacological and optogenetic experiments are needed to explore the functional impact of the learning-associated pause in firing of cholinergic striatal interneurons.

### Future Perspectives

Our data begin to characterize cell type-specific changes in DLS accompanying reward-based learning of a simple goal-directed sensory-to-motor transformation. In future experiments, it will be important to examine changes in synaptic transmission ideally through longitudinal recordings of specific pathways from various cortical and subcortical brain regions onto the distinct cell types of the whisker-related DLS and to assess contributions from diverse neuromodulatory signals in order to gain a more mechanistic understanding of how DLS might contribute to the reward-based learning of this whisker-dependent detection task.

### Data and Software Availability

The complete dataset and Matlab analysis code is freely available at the Open Access CERN database Zenodo with doi: 10.5281/zenodo.5497566 and hyperlink: https://doi.org/10.5281/zenodo.5497566.

## Supplementary Material

zqab049_Sippy_Supplementary_20210909
