# Supplementary material for: Cell Type-Specific Membrane Potential Changes in Dorsolateral Striatum Accompanying Reward-Based Sensorimotor Learning
Source: Function (Oxf). 2021 Sep 21;2(6):zqab049. doi: 10.1093/function/zqab049 (PMC8788857; doi:10.1093/function/zqab049)
Supplement: zqab049_Sippy_Supplementary_20210909 [file zqab049_sippy_supplementary_20210909.pdf]

Supplementary Information

Cell type-specific membrane potential changes in dorsolateral striatum accompanying reward-based sensorimotor learning

Tanya Sippy, Corryn Chaimowitz, Sylvain Crochet and Carl Petersen

The supplementary information consists of:

Supplementary Figure 1, related to Figure 2
Supplementary Figure 2, related to Figure 2
Supplementary Figure 3, related to Figure 3
Supplementary Figure 4, related to Figure 3
Supplementary Table 1

Supplementary Figure 1

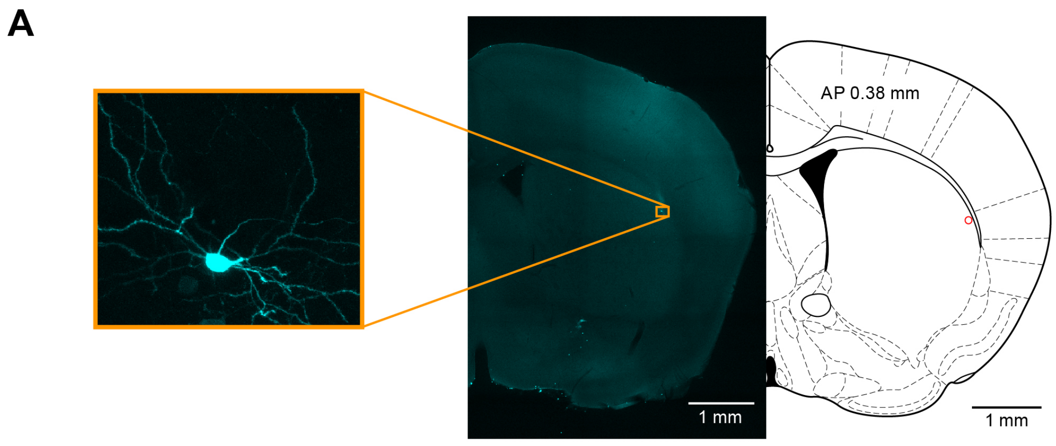

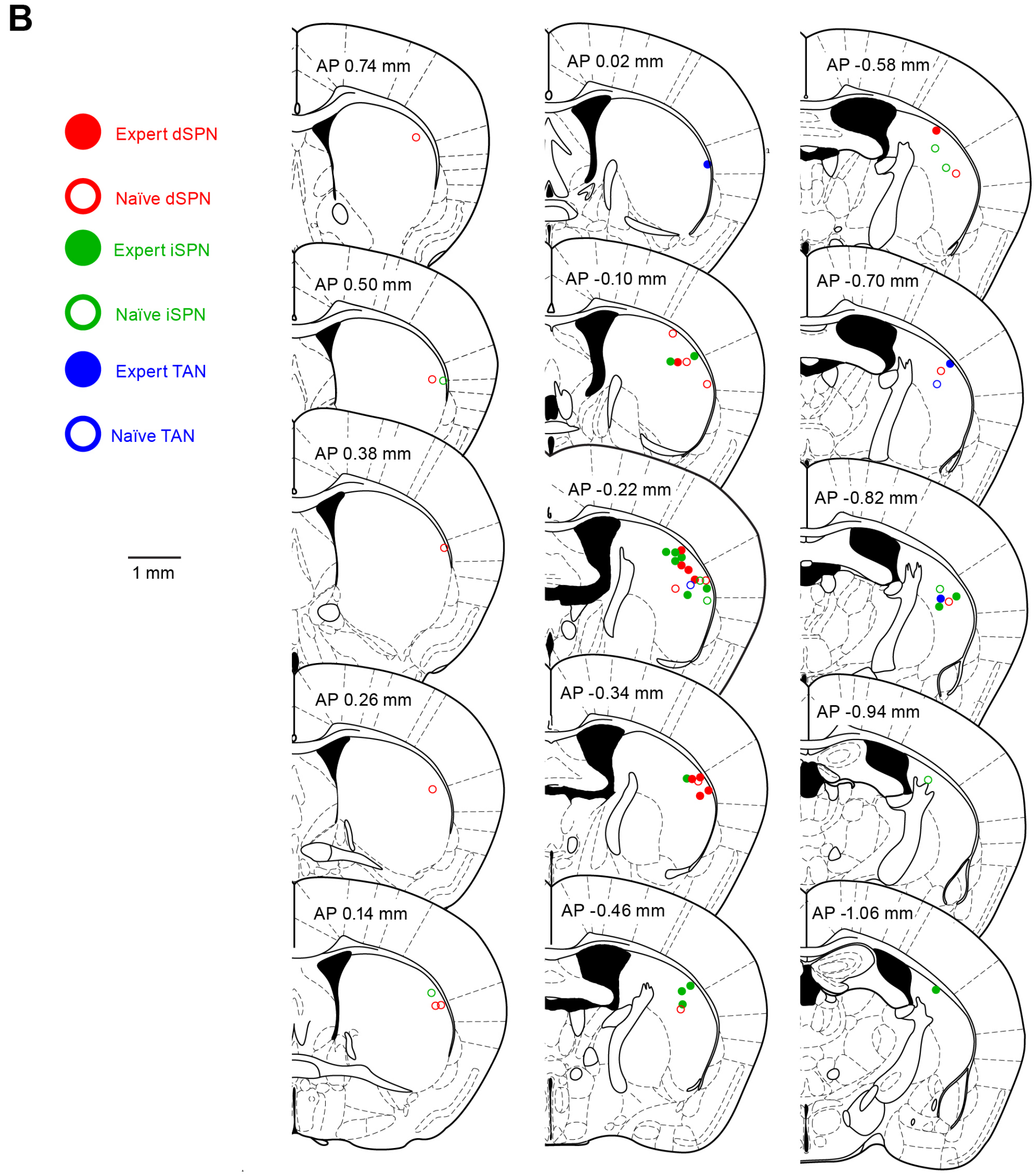

Supplementary Figure 1. Cell locations, Related to Figure 2.

(A) An example biocytin-labelled neuron imaged using confocal microscopy (left). The location of the cell body was superimposed upon a drawing of the best-matching coronal section from a standard mouse brain atlas (Paxinos & Franklin, 2001).

(B) The locations of all biocytin-labelled neurons superimposed upon drawings of coronal sections (Paxinos & Franklin, 2001). Neurons were identified as dSPNs (n = 29 cells, red) and iSPNs (n = 20 cells, green) based on the expression of genetically-encoded fluorescent proteins. TANs (n = 5 cells, blue) were identified based on anatomical and electrophysiological markers. Neurons recorded from naïve mice are indicated by open circles; neurons recorded from expert mice are indicated by closed circles. The somata of the dSPNs, iSPNs and TANs recorded across naïve and expert mice were on average located in a similar region of the DLS. Mean coordinates in mm relative to bregma (mediolateral, ML; anteroposterior AP, dorsoventral DV):

dSPN expert	ML: 2.75	AP: -0.27	DV: 2.42	(n = 12)
dSPN naïve	ML: 2.72	AP: -0.13	DV: 2.59	(n = 17)
iSPN expert	ML: 2.62	AP: -0.43	DV: 2.47	(n = 13)
iSPN naïve	ML: 2.78	AP: -0.27	DV: 2.60	(n = 7)
TAN expert	ML: 2.83	AP: -0.50	DV: 2.69	(n = 3)
TAN naïve	ML: 2.72	AP: -0.46	DV: 2.75	(n = 2)

The locations of dSPNs and iSPNs were not significantly different (MANOVA, Hotelling's trace test statistic, p = 0.21). Because of the small number of anatomically-identified TANs, these were not included in the statistical test.

Supplementary Figure 2

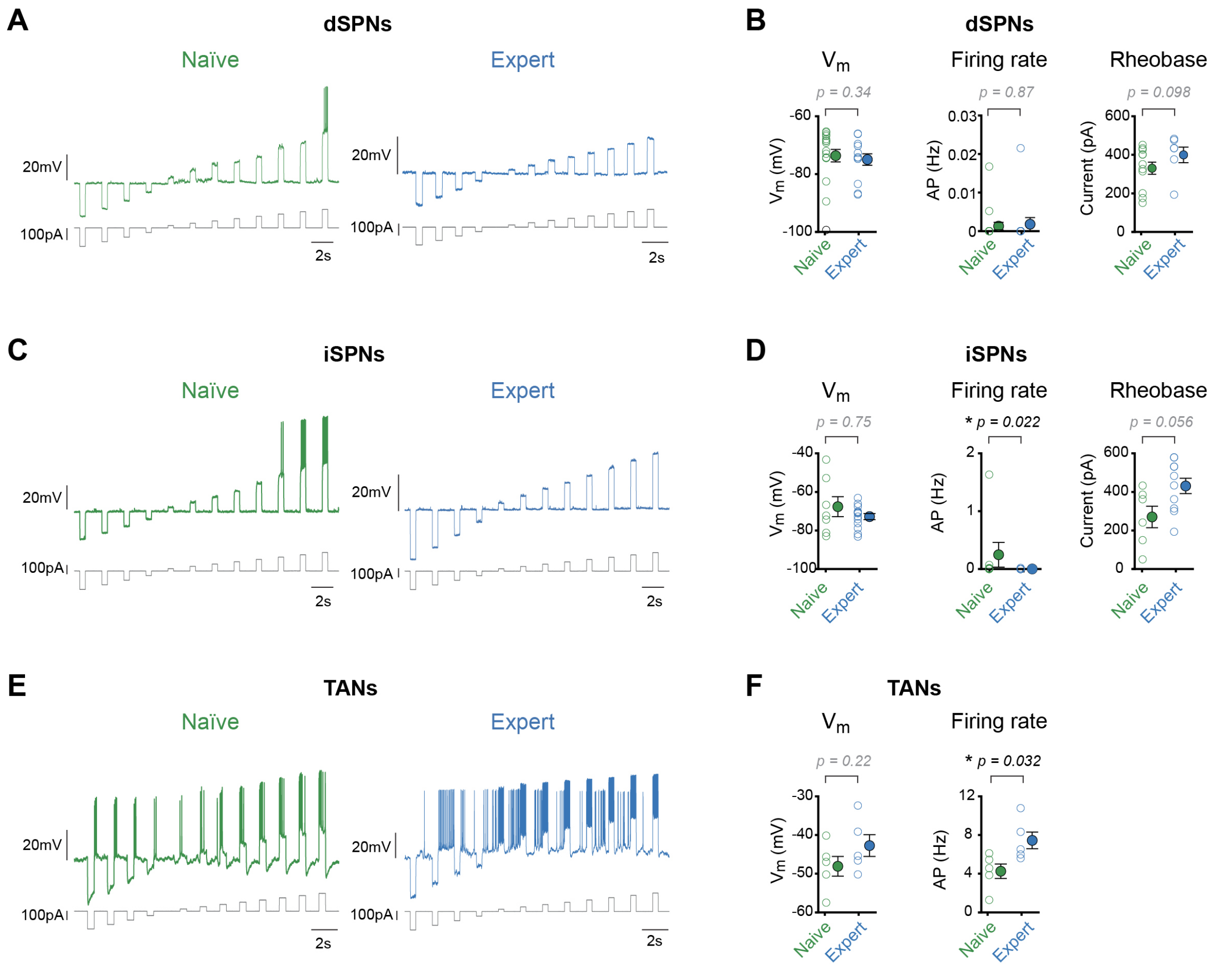

Supplementary Figure 2. Distinct intrinsic electrophysiological properties of SPNs and TANs across naïve and expert mice, Related to Figure 2.

(A) Negative and positive current pulses of different amplitudes (grey traces) were injected through the recording pipette while monitoring the V_m response of example dSPNs recorded in naïve (green) and expert (blue) mice.

(B) Comparison of baseline V_m, baseline action potential firing rate, and rheobase for dSPNs recorded in naïve vs expert mice. Open circles indicate individual cells, closed circles with error bars indicate mean ± SEM; *p* value Wilcoxon-Mann-Whitney test.

(C) As for panel A, but for iSPNs.

(D) As for panel B, but for iSPNs

(E) As for panel A, but for TANs.

(F) As for panel B, but for TANs. Because TANs are spontaneously active, we did not compute rheobase.

Supplementary Figure 3

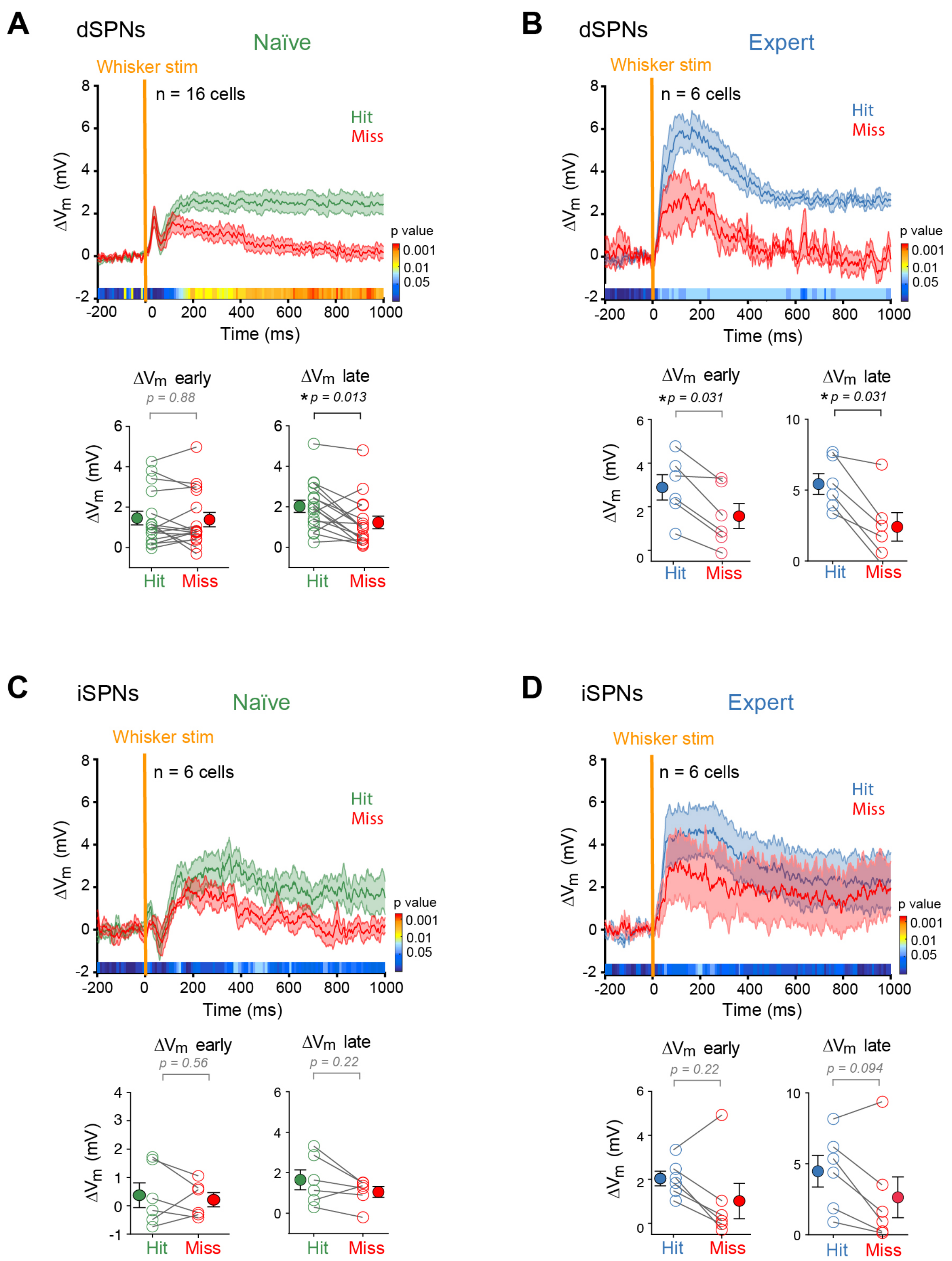

Supplementary Figure 3. Hit vs Miss trials across naïve and expert mice, Related to Figure 3.

(A) Top: Whisker stimulus-triggered ΔV_m average for all hit (green) versus all miss (red) trials recorded from dSPNs in naïve mice. The color-coded bar indicates p values for the difference between hit and miss trials in 10 ms time windows (Wilcoxon-signed-rank test). Bottom: The early ΔV_m (20-50 ms after stimulus) and the late ΔV_m (50-250 ms after stimulus) in naïve dSPNs for hit vs miss (Wilcoxon-signed-rank test). Open circles connected with grey lines are individual cells. Closed circles show mean ± SEM.

(B) Top: Whisker-stimulus triggered ΔV_m average for all hit (blue) versus all miss trials (red) recorded from dSPNs in expert mice. The color-coded bar indicates p values for the difference between hit and miss trials in 10 ms time windows (Wilcoxon-signed-rank test). Bottom: The early ΔV_m (20-50 ms after stimulus) and the late ΔV_m (50-250 ms after stimulus) for expert dSPNs hit vs miss (Wilcoxon-signed-rank test). Open circles connected with grey lines are individual cells. Closed circles show mean ± SEM.

(C) Same as A, but for iSPNs in naïve mice.

(D) Same as B, but for iSPNs in expert mice.

Supplementary Figure 4

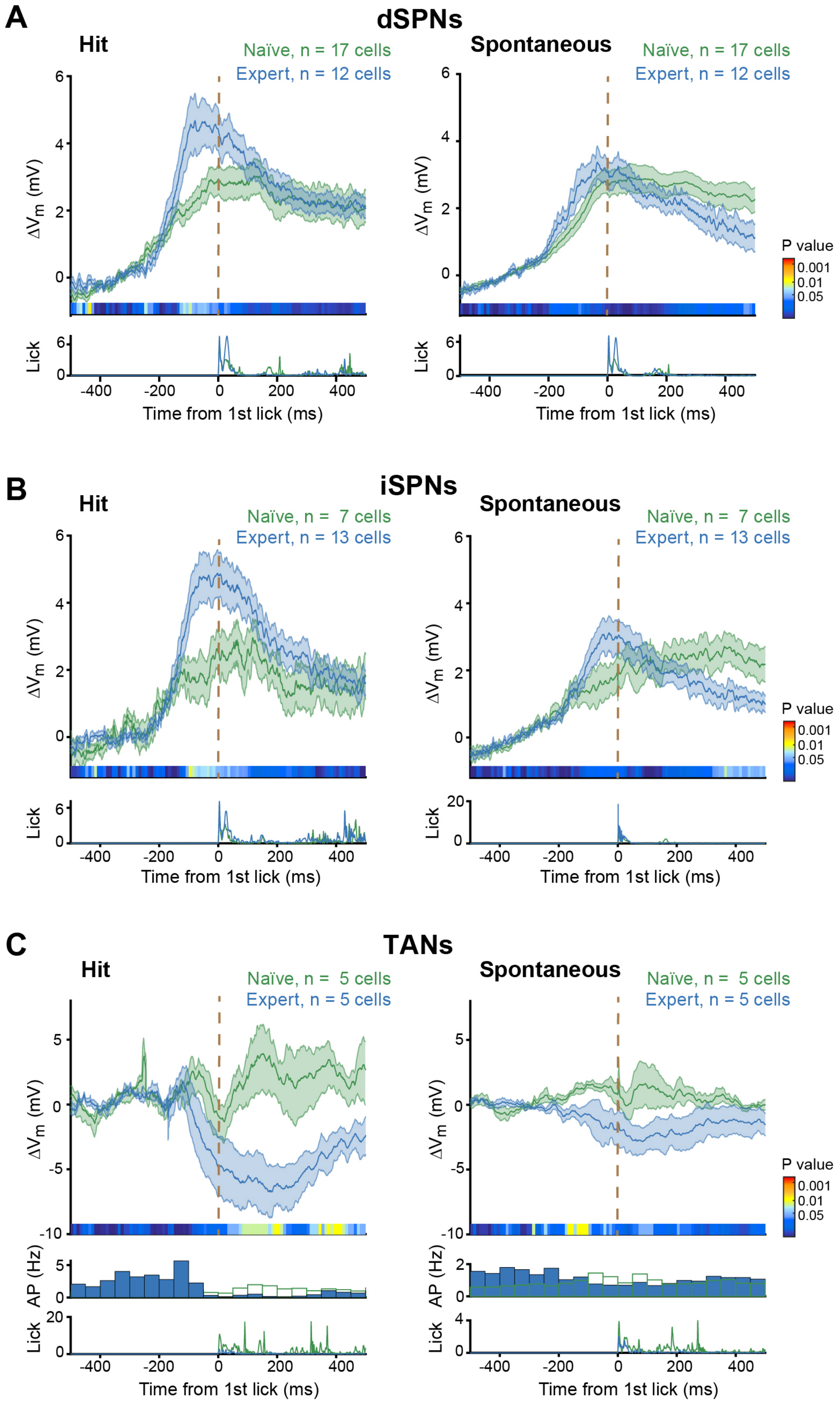

Supplementary Figure 4. Hit trial and spontaneous lick-triggered changes in V_m across naïve and expert mice for dSPNs, iSPNs and TANs, Related to Figure 3.

(A) Left: First lick-triggered ΔV_m average for dSPNs for hit trials in naïve (green) and expert (blue) mice. Right: First lick-triggered ΔV_m average for spontaneous licking in naïve (green) and expert (blue) mice. The color-coded bar indicates p values for the difference between expert and naïve mice in 10 ms time windows (Wilcoxon-Mann-Whitney test).

(B) Same as (A), but for iSPNs.

(C) Same as (A), but for TANs.

Supplementary Table 1

Cell Type	Naïve: number of neurons	Naïve: number of mice	Expert: number of neurons	Expert: number of mice
dSPNs	17	17	12	11
iSPNs	7	6	13	9
TANs	5	4	5	5
Unidentified	20	18	32	25
Totals	49	42	62	48

Supplementary Table 1. Table indicating the numbers of mice and neurons in different experimental groups. Unidentified neurons were not included in our analyses. We typically recorded from one neuron per mouse in order to facilitate anatomical identification. Expert and naïve mice were non-overlapping. Although TANs only represent 1-2 % of the total number of neurons in the striatum, in our blind whole-cell recordings we identified 10 TANs out of the total 111 neurons, thus forming ~10 % of our data set. We think the large size of the cell body of TANs facilitates in vivo whole-cell recording of this neuron type, thus making it more prevalent in our data set than expected.
